# Rethinking energy in parkinsonian motor symptoms: a potential role for neural metabolic deficits

**DOI:** 10.3389/fnsys.2014.00242

**Published:** 2015-01-06

**Authors:** Shinichi Amano, Deborah Kegelmeyer, S. Lee Hong

**Affiliations:** ^1^Department of Biomedical Sciences, Ohio UniversityAthens, OH, USA; ^2^Ohio Musculoskeletal and Neurological Institute, Ohio UniversityAthens, OH, USA; ^3^Division of Physical Therapy, College of Medicine, The Ohio State UniversityColumbus, OH, USA

**Keywords:** Parkinson’s disease, motor deficits, neural metabolic deficits, mitochondrial dysfunction, behavioral inflexibility

## Abstract

Parkinson’s disease (PD) is characterized as a chronic and progressive neurodegenerative disorder that results in a variety of debilitating symptoms, including bradykinesia, resting tremor, rigidity, and postural instability. Research spanning several decades has emphasized basal ganglia dysfunction, predominantly resulting from dopaminergic (DA) cell loss, as the primarily cause of the aforementioned parkinsonian features. But, why those particular features manifest themselves remains an enigma. The goal of this paper is to develop a theoretical framework that parkinsonian motor features are behavioral consequence of a long-term adaptation to their inability (inflexibility or lack of capacity) to meet energetic demands, due to neural metabolic deficits arising from mitochondrial dysfunction associated with PD. Here, we discuss neurophysiological changes that are generally associated with PD, such as selective degeneration of DA neurons in the substantia nigra pars compacta (SNc), in conjunction with metabolic and mitochondrial dysfunction. We then characterize the cardinal motor symptoms of PD, bradykinesia, resting tremor, rigidity and gait disturbance, reviewing literature to demonstrate how these motor patterns are actually energy efficient from a metabolic perspective. We will also develop three testable hypotheses: (1) neural metabolic deficits precede the increased rate of neurodegeneration and onset of behavioral symptoms in PD; (2) motor behavior of persons with PD are more sensitive to changes in metabolic/bioenergetic state; and (3) improvement of metabolic function could lead to better motor performance in persons with PD. These hypotheses are designed to introduce a novel viewpoint that can elucidate the connections between metabolic, neural and motor function in PD.

## Background and introduction

Parkinson’s disease (PD) is characterized as a chronic and progressive neurodegenerative disorder that results in a variety of debilitating symptoms. The cardinal motor symptoms of PD include bradykinesia, resting tremor, rigidity, and postural instability. Research spanning several decades has emphasized dysfunctions of basal ganglia, predominantly resulting from dopaminergic (DA) cell loss, as the primarily cause of the aforementioned parkinsonian features.

**Figure 1:**
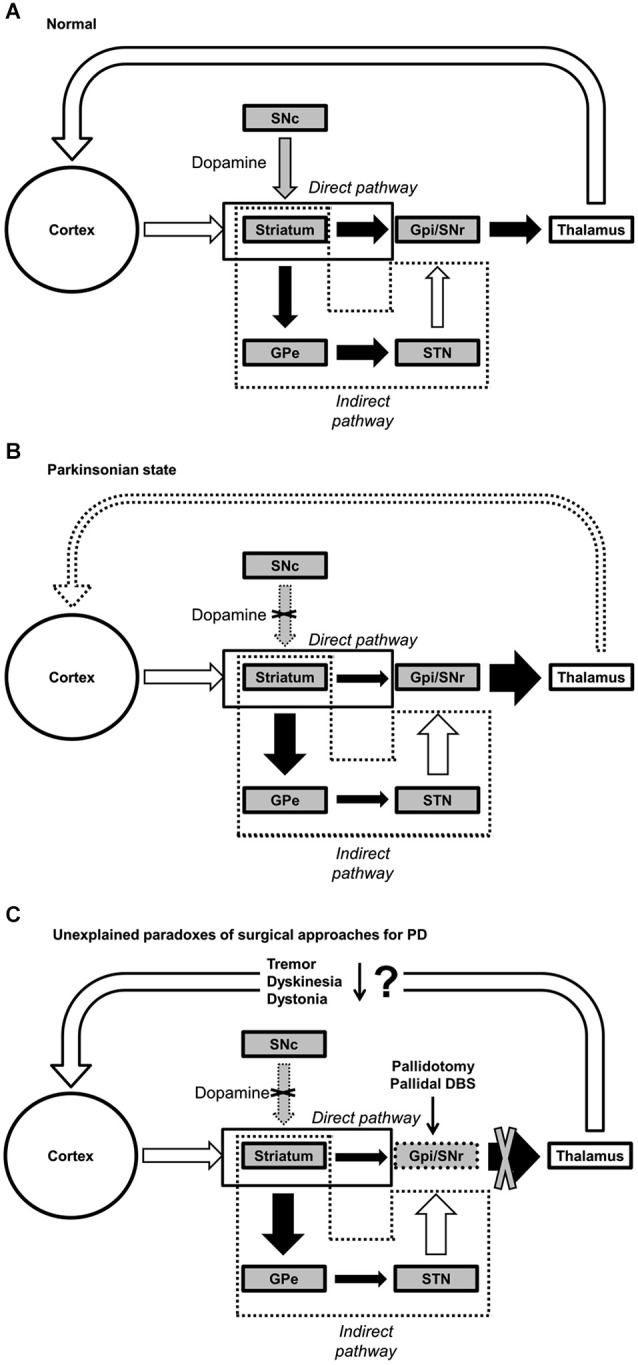
**Functional connectivity within the basal ganglia. (A)** Normal, **(B)** Parkinsonian state, and **(C)** paradoxical outcomes of pallidotomy and pallidal DBS for PD. Black, white, and gray arrows represent inhibitory, excitatory and dopaminergic (DA) transmissions, respectively. *Abbreviations*: GPe, globus pallidus externus; GPi, globus pallidus internus; SNc, substantia nigra pars compacta; SNr, substantia nigra pars reticulata; STN, subthalamic nucleus; DBS, deep brain stimulation.

Classical pathophysiological model of PD, namely the “rate” model (or two pathway model) (Albin et al., 1989; DeLong, 1990; Wichmann and Delong, 1998; Wichmann et al., 2011), suggests that abnormal activation or excessive inhibition of cortico-basal ganglia-cortical loops resulting from changes in neuronal firing patterns in the output channel of basal ganglia (i.e., substantia nigra pars reticulata (SNr) and globus pallidus pars interna (GPi)) is an essential feature of PD. The basal ganglia are a group of interconnected subcortical nuclei, and they connect with cerebral cortex, thalamus and other areas of the brain to facilitate various motor functions (Albin et al., 1989). In the classic rate model, there are two distinct pathways: direct and indirect pathways, each of which appears to play a specialized role, and this is the concept central to this classic model (Figure 1).

Briefly, while the direct pathway facilitates the desired motor program for execution of the selected action by exciting the cortex, the indirect pathway inhibits competing and unwanted motor programs to efficiently execute the desired movement (Albin et al., 1989; DeLong, 1990; Mink, 1996; Figure 1A).

Dopaminergic neuronal loss in the substantia nigra pars compacta (SNc) affects both pathways differently, resulting in decreased inhibitory inputs (via the direct pathway) and increased excitatory inputs (via indirect pathway) to the GPi. As a whole system, the rate model posits that dopamine depletion would lead to an increase in tonic inhibition of thalamus and a reduction in excitatory drive to motor cortex (Fearnley and Lees, 1991; DeLong and Wichmann, 2007; Figure 1B). Therefore, it is often thought that individuals with PD often manifest subsequent reduction in movement (e.g., akinesia, hypokinesia, and bradykinesia), as a consequence of the aforementioned dysfunction of basal ganglia (Albin et al., 1989; Vingerhoets et al., 1997; Majsak et al., 1998; Lozza et al., 2002). Although this classic model is widely accepted, it does not provide rational explanations for some hyperkinetic features of PD, including but not limited to dystonia, levodopa-induced dyskinesia, tremor, and rigidity (Marsden and Obeso, 1994; Obeso et al., 2000, 2008; Montgomery, 2007; Brown and Eusebio, 2008; Rodriguez-Oroz et al., 2009).

The central tenet of the rate model is that increased GPi discharge rate is at the core of PD, but a growing body of literature has shown that GPi output rates in PD at rest are not significantly different than the rate in controls in either MPTP (Goldberg et al., 2002; Soares et al., 2004) or non-MPTP (Filion, 1979; Percheron et al., 1993) animal models. The model further suggests that increased inhibition to thalamo-cortical projections reduce neuronal activity in motor cortex, leading to a reduction in motor behavior. However, many animal model studies refute this postulation (Doudet et al., 1990). Furthermore, comorbidity of hyper- and hypo-kinetic motor feature of PD cannot be explained by the rate model because of its emphasis on explaining reduced motor behavior. Perhaps, one of the major challenges to the rate model is the effects of a pallidotomy (i.e., surgical lesion to the GPi). According to the rate model, hyperkinetic movements are the consequence of increased inhibition to the GPi by the indirect pathway and resultant decreased inhibition of the thalamus, leading to the unwanted and excessive movements. Contrary to the rate model, pallidotomy alleviates hyperkinetic features such as tremor (Laitinen et al., 1992; Taha et al., 1997), dystonia (Vitek and Bakay, 1997) and levodopa-induced dyskinesia (Lozano et al., 1995; Lang et al., 1997; Figure 1C). Effectively, these inconsistencies have driven researchers to explore alternative models, such as oscillator systems theory (Montgomery, 2007). In sum, the classic rate model is not sufficient to fully and comprehensively explain PD. Therefore, it is imperative to seek for an alternative model to answer the one simple, yet big and unanswered, question; why do those particular parkinsonian features manifest themselves? In other words, is the degeneration of DA neurons directly related to particular parkinsonian features? Or, are the motor symptoms of PD an indirect consequence of long-term adaptation in response to these neuronal losses?

While PD is generally viewed from the perspective of a neurological disorder, there is now evidence that PD should also be associated with metabolic dysfunction. Indeed, weight loss is a well-known feature of PD (Kashihara, 2006), affecting more than half of PD patients (Abbott et al., 1992). Intriguingly, this weight loss in persons with PD appears to begin several years prior to initial diagnosis, despite increased dietary intake (Davies et al., 1994; Chen et al., 2003). Another possible sign of PD prior to its onset is aggregation of alpha synuclein in the colon (Shannon et al., 2012). One might not consider the buildup of alpha synuclein in the colon as a direct metabolic problem, however, problems with feeding, bowel movements, and/or nutrient absorption would indirectly contribute to energy deficits including a shortage of metabolic substrate and reduced energy intake. Furthermore, exaggerated susceptibility to fatigue even at lower power outputs and elevated VO_2_ consumption during cycling exercise regardless of power output demand is observed in PD patients when compared to healthy elderly counterparts (Protas et al., 1996; Stanley et al., 1999; Christiansen et al., 2009).

The role of dopamine and energy metabolism in brain function is intertwined. Schwartz et al. (1976) found that dopamine lesions result in reduced glucose uptake in the forebrain, indicating that dysfunctional DA neurotransmission leads to reduced substrate availability for neural metabolism. In addition, dopamine itself is related to glucose metabolism and insulin sensitivity (Luo et al., 1997; Jetton et al., 2001), and thus, the loss of dopamine neurons in PD is likely to contribute to dysfunctional glucose metabolism. Interestingly, deficits in the availability of metabolic substrate in the brain negatively affect dopamine neurons. Chan et al. (1994) observed that a sudden loss of adenosine triphosphate (ATP) levels in striatum led to the loss of dopamine neurons. A growing body of literature documents this vulnerability of DA neurons to energy deficits (Brouillet et al., 1993, 1995; Zeevalk et al., 1995, 1997, 1998). Collectively, these findings demonstrate that metabolic deficits in the brain lead to the loss of dopamine function and vice versa.

A growing body of literature argues that mitochondrial dysfunction is a common physiological feature in PD and is causing aggravated metabolic dysfunction (Chan and Mcquibban, 2013; Gaweda-Walerych and Zekanowski, 2013a,b; Lehmann and Martins, 2013). Without question, metabolic energy is essential to maintain the function of various organs and systems of the human body. Chief among them, the brain requires a significant proportion of total body energy expenditure, which is approximately 20–25% of total energy (Mink et al., 1981; Attwell and Laughlin, 2001; Magistretti and Allaman, 2013). A reduction in energy supply due to metabolic dysfunction, along with increased brain energy costs due to neural communication deficits (see Hong and Rebec (2012)), could be a central problem in PD that has not yet received sufficient discussion. Metabolic deficits also raise the possibility that the high metabolic demand in PD could arise from DA neurons since DA neurons give rise to a large number of synapses in the central nervous system (CNS).

The goal of this paper is to develop a theoretical framework that parkinsonian motor features are behavioral consequence of a long-term adaptation to their inability (inflexibility or lack of capacity) to meet energetic demands, rather than being solely a manifestation of dysfunction or deficiency of neural circuitry. We will also develop hypotheses that can be tested based on the overarching theory that PD motor symptoms are associated with neural metabolic deficiencies.

## Behavioral inflexibility in Parkinson’s disease

We often characterize PD as a set of distinct problems: slowness, reduced movement, and tremor. Yet, when taken as a whole, PD is truly a paradox where we observe both more and less movement at the same time. When both the hyper- and hypo-kinetic consequences of PD simultaneously, the disorder leads to a state where: (1) when the person wants to move, he/she is stuck (bradykinesia, akinesia, and freezing); and (2) when the person wants to stay still, he/she continues to move (tremor). Essentially, the neuromotor system in PD suffers from a problem of *inflexibility*, where the potential functional range of behaviors that it can engaged in has been restricted. Examples of inflexibility within the context of aging is a reduced ability to generate both highly variable and highly repetitive behaviors (see Vaillancourt and Newell (2002) for a review) and an inability to control both high and low muscle force outputs.

What then ensues is a question of the potential source of inflexibility in PD. In our recent position paper on neural noise and behavioral variability in aging, we proposed that a restriction of metabolic capacity could be a central problem that restricts flexibility in neural activation (Hong and Rebec, 2012). Effectively, without sufficient metabolic fuel to both manage background noise and increase firing frequency, the brain is left with a narrowed functional range. Perhaps, the same is true in PD, but at a far more extreme level than observed in aging, where metabolic problems are key components that shape PD symptoms. The following sections review metabolic deficits in PD.

## Mitochondrial dysfunction in Parkinson’s disease

Mitochondrial dysfunction has been implicated as a cause of PD (Abou-Sleiman et al., 2006; Banerjee et al., 2009). One of the primary functions of mitochondria is to produce ATP through the electron transport chain. The majority of energy consumed in cellular activities is produced either directly or indirectly by mitochondria. Thus, maintaining mitochondrial function is vital for cell survival and thus strongly associated with both aging and neurodegeneration like PD.

The association between aging and PD are once again gaining attention and new investigations are being conducted. Furthermore, of a large number of risk factors of PD previously proposed, one of the primary risk factors is aging. In fact, the average age of onset is estimated at approximately 60–65 years (Twelves et al., 2003). Approximately 1–2% of the population over age of 65 is affected by this disease, and the proportion increases with age. As a result, a growing body of literature proposes that aging and PD could be related at the level of cellular mechanisms, where the cell loss occurring with PD is more accelerated, exaggerated, and region-specific (Collier et al., 2011; Bolam and Pissadaki, 2012; Cai et al., 2012; Pissadaki and Bolam, 2013). Indeed, both aging and PD share one progressive cellular change: mitochondrial dysfunction.

A growing body of evidence suggests that mitochondria interact with many proteins, including alpha-synuclein, parkin, and PINK1, which are also associated with formation of PD. Also, there appears to be an association between mitochondrial function and parkinsonian features since the neurotoxins used to generate animal models of PD, 6-Hydroxydopamine (6-OHDA) and 1-methyl-4-phenyl-1,2,3,6-tetrahydropyridine (MPTP; Langston and Ballard, 1983; Schober, 2004) affect mitochondria and replicate most of the cardinal symptoms of PD (with the exception of Lewy body formation). Overall, these lines of evidence present a view of PD as a process of accelerated decline in the later stages of life, above and beyond normal age-related deterioration of function.

While the efficiency of energy production appears to be reduced in aging due to the aforementioned mitochondrial dysfunction, aging, in fact, also results in an increase in energy expenditure for activities of daily living as well. For example, Martin et al. (1992) showed that the aerobic demands for walking, which was normalized to body mass and walking speed, was 8% higher in elderly individuals than their young counterparts. Higher energy expenditure during submaximal movement tasks in elderly individuals has also been consistently reported (Grimby and Soderholm, 1962; Waters et al., 1983; Larish et al., 1988). Given that muscle mass and strength decrease with advancing age, elderly individuals are likely to face behavioral inefficiencies which require them to recruit more motor units to produce sufficient force to meet a given task demand (e.g., speed, steadiness, etc.) in comparison with younger adults (Martin et al., 1992). Moreover, at the neural level, energy costs would also be increased due to a greater need for noise reduction and increased firing frequency (Hong and Rebec, 2012). Thus, decreased muscular, behavioral, and neural efficiency due to aging could lead to compensatory adaptations in response to the increased energy demands in order to maintain metabolic homeostasis (Schrack et al., 2010). Effectively, PD is a situation where there are high energetic demands that are accompanied by reduced energy supply (i.e., reduced substrate availability), prompting an energy-conserving response.

## Neurophysiological adaptation to energetic/metabolic deficits in Parkinson’s disease

Although the exact etiology of mitochondrial dysfunction in PD still remains to be defined, it is a pivotal problem in PD. Furthermore, the impact of such metabolic deficits on brain and behavior are yet to be widely explored. However, the human organ requiring the largest amount of energy is the brain, and CNS deficits are central to the symptoms of PD. Specifically, humans devote about 20–25 % of basal metabolism to the brain (although it only weighs only 2% of total body weight), and most of the energy consumed in the brain is to maintain electrical activity in neurons (Mink et al., 1981; Magistretti and Allaman, 2013). While maintaining resting potential requires a relatively small amount of energy (13% of total energy), almost half of total energy (43%) is expended to fire action potentials (Attwell and Laughlin, 2001). Moreover, energy costs of neuronal firing increase as a function of firing frequency (Attwell and Laughlin, 2001).

Higher firing frequencies not only demand more metabolic energy, they demand greater firing precision as well, essentially due to a reduced “margin for error” in terms of neuronal information transmission. Deviations or errors in neural activities are an inherent phenomenon in the brain, as random background activity or neural noise is pervasive at all levels of the CNS (see Faisal et al. (2008) for a review). Based on experimental and mathematical modeling, Tsubo et al. (2012) has demonstrated that neurons attempt to maximize the amount of information (entropy) while minimizing energy cost and noise on neuronal firing patterns, namely conditional maximization firing-rate entropy hypothesis (CMFE). In other words, neurons seek to find an optimal frequency distribution of neuronal firing to maximize information transmittal while suppressing energy cost and neural noise (Hong and Rebec, 2012). Simply, noise reduction comes at a metabolic cost.

Increased beta oscillation in PD could be another reflection of a compensatory strategy for energy deficits. Oscillatory activity can lead to more efficient temporal summation on target neurons if it is constrained in a spatially discriminated scale, while it might also potentially reduce information encoding capacity (indexed by entropy) if it occurs across large populations of neurons (Uhlhaas et al., 2010; Little and Brown, 2014). The increased beta activity could occur by replacing high frequency firing patterns (energetically inefficient) with lower frequency oscillatory activities. Effectively, the frequency distribution is likely compressed toward lower frequency bands (e.g., pushing γ to β, β to α), improving the signal-to-noise ratio and reducing overall energy cost at the expense of the amount of information that can be transmitted.

Similar to the aforementioned neuronal adaptations observed at the neurophysiological level of CNS, it is possible that there are changes at the cellular (structural) level. Selective degeneration of DA neurons within the SNc is considered to be one of the major neurological complications associated with principal parkinsonian features (Bolam and Pissadaki, 2012). Based on this conceptual framework, the loss of DA neurons in the SNc disrupts normal function of basal ganglia circuitry, resulting in inadequate activation of desired motor programs and excessive inhibition of undesired and competing programs (di Michele et al., 2013). However, the causal relationship between DA neurodegeneration and motor impairments in PD remains unclear. In other words, is there a direct link between loss of DA neurons and specific parkinsonian features? Is the neurophysiological change observed in PD just a consequence of long-term behavioral adaptations?

Research has begun to elucidate why DA neurons in the SNc are exceptionally vulnerable and selectively and exceptionally degenerated. A series of previous research has suggested that the extreme energy cost is required to structurally and functionally maintain the DA neurons in SNc because of the large number of synapses of the these neurons when compared to other regions and types of neurons. Specifically, the number of synapses from a single DA neuron of SNc in humans was recently estimated by Bolam and Pissadaki (2012), based on previous PD model studies in rats, which has shown that there are about 12,000 DA neurons in the SNc (Oorschot, 1996; Nair-Roberts et al., 2008). It was estimated that a single DA neuron of the SNc in rats gives rise to approximately 102,165 to 245,103 synapses at the level of striatum. In humans, these synapses would be a few orders of magnitude higher as compared to rats. In terms of other types of neurons in the basal ganglia, the SNc gives rise to much higher numbers of synapses by two orders of magnitude at the very least (Bolam and Pissadaki, 2012). Furthermore, the unique length, exceeding four meters, and complexity of their axons compound bioenergetic deficits and contribute to their selective vulnerability in PD (Pissadaki and Bolam, 2013). Based on these findings, it was proposed that the high metabolic cost of DA neurons makes them the first to face elimination in the face of an energy shortage (Surmeier et al., 2010a,b; Bolam and Pissadaki, 2012).

While more detailed research is necessary, it can be postulated that these neurophysiological changes occurring in the basal ganglia, particularly SNc, can be considered as a necessary adaptation, responding to lack of energy supply and/or their susceptibility due to bioenergetic demand to maintain their functional and structural characteristics. One should also consider that persons with PD are likely to exhibit neurobehavioral inflexibility since the constraint arising from metabolic energy shortage will result in a narrowing of the neural oscillation frequency range. More importantly, the question that then arises is whether these alterations to neural function could give rise to the motor behavioral symptoms in PD.

## Behavioral adaptation as an “energy saving” strategy in Parkinson’s disease

Considering all of the literature reviewed in the paper thus far, one would hypothesize that people with PD will have to adapt their behaviors in order to overcome accelerated and exaggerated energy deficits. This adaptation could occur at various levels: cellular, physiological and behavioral levels, as a means of minimizing energy cost with respect to task, environmental and other constraints (Sparrow and Newell, 1998). Daily energy expenditure is indeed lower in PD patients compared to healthy elderly, primarily due to reduced physical activity (Toth et al., 1997). Conventional wisdom, would suggest that tremor would lead to increased, rather than decreased metabolic cost (Poehlman et al., 1995). Furthermore, alterations to gait in PD are generally considered to be less efficient than normal gait (Pelosin et al., 2009). Both of these factors combined, would lead to weight loss.

Indeed, symptom severity (i.e., increased tremor, rigidity, and hypokinesia) is associated with greater weight loss in persons with PD (Lorefält et al., 2004). Furthermore, a large body of literature reports significant weight loss as an important problem in persons with PD (Abbott et al., 1992; Beyer et al., 1995; Bachmann and Trenkwalder, 2006; Kashihara, 2006). It is important to note that the weight loss occurs regardless of increased energy intake (Davies et al., 1994; Chen et al., 2003; Lorefält et al., 2004) or changes in daily energy expenditure (Delikanaki-Skaribas et al., 2009), or with increased rest (Lorefält et al., 2004). This raises a question as to whether symptom severity is necessarily the cause of the weight loss, or, if it is a response to other underlying metabolic issues that result in weight loss.

Fluctuations in PD motor symptoms, even with levodopa therapy, also raise another possibility that parkinsonian motor features cannot be fully explained by dopamine depletion alone. In a mouse model without metabolic deficits, Sotnikova et al. (2005) demonstrated that PD behavioral phenotype of transient depletion of striatal dopamine can be reversed successfully using levodopa. Yet, PD patients initially respond well to the introduction of levodopa, but face a progressive decline in the benefits of levodopa administration after a few years (Chase, 1998). Assuming dopamine loss is the primary source of parkinsonian motor features, levodopa therapy should be sufficient to restore and stabilize motor function. In other words, symptom severity and its fluctuations throughout the day should be strongly associated with dopamine levels and predictable based on dose and half-life of levodopa, i.e., how much of the drug is given, and how quickly its effects wear off. However, long-term administration of DA therapy, which is supposed to solve dopamine shortage and optimally regulate the dopamine level, cannot fully control motor symptoms, or even worsen them (e.g., motor fluctuation, levodopa-induced dyskinesia and sleep disturbance) (Fahn et al., 2004).

Alterations to metabolic function provide a potential explanation for the variability and continued fluctuations in motor symptoms despite therapy. A good example of such phenomena is a change blood glucose fluctuation patterns that become dysfunctional in people with metabolic syndrome and type II diabetes (Churruca et al., 2008; Costa et al., 2014). While healthy, normoglycemic individuals exhibit rapid, small, and irregular blood glucose fluctuations throughout the day, people with metabolic syndrome and type II diabetes exhibit slow, large, and systematic changes in blood glucose levels. This “loss of complexity” has been observed in aging (Lipsitz and Goldberger, 1992; Goldberger et al., 2002) and a variety of other disorders, including PD (Vaillancourt and Newell, 2002). This is consistent PD symptom fluctuations where there are large, slow, and systematic fluctuations, where “on” periods are very good, while “off” periods lead to severe symptoms. In addition, a body of previous studies suggested that DA levels and motor symptom severity are not strongly correlated (Mouradian et al., 1987, 1988; Fabbrini et al., 1988; Chase, 1998). Given that PD symptoms generally do not appear until a large proportion of DA neurons (~80%) in SNr are degenerated (Marsden, 1990; Lang and Lozano, 1998; Dauer and Przedborski, 2003; Ross et al., 2004), a critical question arises; how can motor fluctuations still occur when a majority of the DA neurons in SNr have been lost? In other words, the limited number of neurons will also limit the potential variation and range of DA levels that can be produced, thus leading to relatively stable symptoms. Metabolic fluctuations provide a potential explanation as to why symptom fluctuations exist, and further declines in metabolic function explain the gradual resistance to levodopa therapy.

**Figure 2:**
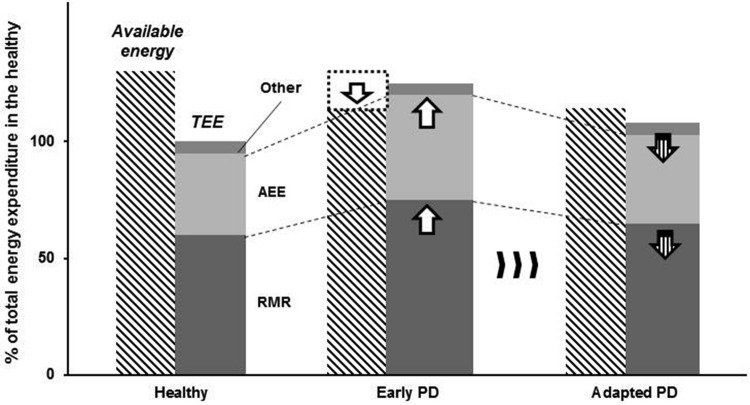
**Schematic illustrating the adaptive strategy in response to energy imbalance in persons with PD**. Persons with PD exhibits reduced energy supply (limited available energy), whereas resting metabolic rate (RMR) and activity energy expenditure (AEE) are increased. As a result, energy depletion would occur without any compensatory adaptations. We propose that parkinsonian motor features are behavioral consequences of long-term adaptation to a neural metabolic deficit in persons with PD.

Here, we propose that the cardinal movement disorders of PD could be the consequence of an adaptive compensatory response to the decreased ability to meet metabolic demands for activities of daily living (Figure 2). For instance, Schrack et al. (2010) suggested that elderly individuals are likely to develop their adaptive behaviors (e.g., reduced walking speed) to conserve their energy since essential energy (i.e., the energy essential for living) is increased with age, resulting in decreased available energy (i.e., the energy available to an individual in addition to the resting metabolic rate (RMR)). Therefore, parkinsonian motor features could possibly be considered as economizing, which is similar to adaptive behaviors observed in elderly individuals (Schrack et al., 2012). Thus, the question that arises is whether it is possible that the motor symptoms of PD could in fact be energy-saving strategies. We will explore this possibility in the subsequent sections.

Bradykinesia, which describes slowness of voluntary movements, is usually one of the most disabling symptoms for persons with PD. Bradykinesia presents in the early stages of the disease, often accompanied by hypophonia (slow speech; Liotti et al., 2003), micrographia (small writing; McLennan et al., 1972; Oliveira et al., 1997), and saccadic hypometria (small eye movements; Choi et al., 2011). Similarly, gait in persons with PD can be characterized as bradykinetic gait with reduced speed and step length (Morris et al., 1996). Although decreased cortical activity as a consequence of altered basothalamic outputs (exaggerated inhibitory outputs toward cortical areas) appears to be associated with bradykinesia, the exact underlying mechanism is unclear (Hallett, 2003; Wichmann and DeLong, 2003). Here, a decrease in cortical activity would also be consistent with energy conservation at the neural level, reducing the number of action potentials needed.

Behaviorally, micrographia can be improved when visual cues to write larger (lined paper) are provided to persons with PD, i.e., when cued them to write larger (McLennan et al., 1972). This phenomenon is consistent with another previous finding that persons in PD are capable of walking just as fast as their age-matched counterparts, but are less able to maintain the target speed for prolonged periods of time (see Lim et al. (2005) for a review). Essentially, the physical capacity (for lack of a better phrase) to achieve motor task demands remains in place. These studies indicate that persons with PD seem to function at a lower proportion of their maximal capacity. This suggests that the reduced movement speed observed in individuals with PD do not necessarily arise from an inability to perform the movements *per se*.

This leaves the question of what potential internal mechanisms might be prohibitive of the performance of fast movements. From a muscle activation standpoint, bradykinesia has the benefit of demanding the recruitment of slower motor units, reducing the demand for activation of the more energy expensive type II or fast-twitch fibers (Bottinelli et al., 1994; Zierath and Hawley, 2004; Schiaffino and Reggiani, 2011). For the purpose of voluntary movements, an endpoint always exists at the termination of an actions and/or the preparation of subsequent action plans. For instance, when people reach to an object to grasp, there are a series of motor tasks to complete: initiation of reaching movement, acceleration-deceleration of the arm, preparation of grasping (creating hand aperture prior to a grasping action). The ultimate goal of this reaching task is to minimize deviations from the optimal final position needed to grasp an object efficiently, not necessarily to minimize the duration to reach the object, under the assumption that neural noise is signal-dependent (Harris and Wolpert, 1998). As a result, moving faster is energetically more expensive as large control signals consume more energy for action potentials (Attwell and Laughlin, 2001) and noise reduction (Tsubo et al., 2012).

There is an additional energetic benefit for moving slowly in order to minimize undesired movement variability that interferes with task performance. The neuromotor system is thought to attempt to keep adjustments to task-related motor variance to a minimum, hence the term “minimum intervention principle” (Todorov and Jordan, 2002; Todorov, 2004). Effectively, task-irrelevant behavioral variability is generally disregarded as long as it does not affect the task goal. This also means, however, that faster movements will result in larger errors that in turn will require larger corrective movements, i.e., larger muscle contractions. Thus, these converging concepts raise the possibility that motor slowing is a compensatory adaptation to minimize task-relevant variability, and this adaptation is manifests itself as bradykinesia in PD.

### Gait disturbance

Much like the problem of bradykinesia, Parkinsonian gait is also hypokinetic, which can be characterized by spatiotemporal disturbance compared to neurologically healthy individuals. Even in normal aging, seniors tend to walk more slowly to compensate for the increased energy cost of walking (Schrack et al., 2012). Persons with PD often walk slower with greatly reduced step length, accompanied by slowed and decreased arm swing (for a review, see Amano et al., 2013). Although neurodegeneration is believed to be related to the impairment in functional motor tasks, energy cost of gait performance could also critically contribute to its decline since preferred level of energy cost is likely to be associated with speed of movement (Taylor, 1985; Waters et al., 1988). Indeed, previous research showed that the preferred gait frequency is self-optimized and could be predicted by the resonance properties of the limb so that work executed by muscles and the energetic requirements to maintain gait can be minimized (Holt et al., 1991). Simply, walking slower or faster than the natural speed “prescribed” by one’s body proportions leads to a higher metabolic cost.

Gait is a complex motor task that includes both active and passive control mechanisms, according to previously-reported mathematical modeling (McGeer, 1990; Bauby and Kuo, 2000; Kuo, 2007). While forward propulsion can be maintained with little actuation and no active adjustments, lateral (side-to-side) stability cannot be achieved without active sensorimotor control (Bauby and Kuo, 2000; Donelan et al., 2001). This active lateral balance control in the mathematical walking model can be compatible with PD gait since they have more difficulty in maintaining lateral stability rather than forward propulsion (Van Wegen et al., 2001; Horak et al., 2005; King and Horak, 2008).

Reduced step length and an increase in double-limb support time, both characteristic of PD gait, are beneficial for stability, leading to altered biomechanical gait pattern. Superficially, this compensatory gait strategy would seem to require more energy, and indeed, previous studies suggest that this abnormal gait pattern leads to poorer economy of gait (i.e., higher sub-maximal VO_2_ /VO*_2max_*) in persons with PD when compared to age-matched healthy individuals (Christiansen et al., 2009; Katzel et al., 2012). It is especially important to note here that Katzel et al. (2012) calculated gait economy based on measured VO_2_ as a proportion of predicted VO_2_ using the American College of Sports Medicine (ACSM) equation. The ACSM equation, however, only takes walking speed into account and does not include height or body mass in the calculations. What this does is make an assumption that persons with PD walk at any given speed with similar biomechanics to healthy controls. Moreover, at the same absolute speed, the person with PD would be walking at a higher relative speed (i.e., higher percentage of maximum) than an age-matched control. As a result, it is not yet certain whether or not the aforementioned biomechanical changes in gait pattern of persons with PD primarily result in elevated energy requirements during walking. These altered spatiotemporal gait parameters are simply end products of a multifactorial motor task comprising precise neural motor commands, efficient force productions and joint motion control, and adequate postural stability against any postural threats, all of which could burden the energy requirement of gait.

If considered more deeply, the PD gait pattern is actually a means of making a slow and low amplitude gait pattern more energy efficient. The stooped posture often observed in persons with PD is a critical factor. This stooped or hunched posture enables persons with PD to lower their center of mass (COM). This is based on the premise that minimizing the displacement of COM as much as possible is the best way to achieve economical gait. Moreover, the shift in whole body COM alters the individual’s preferred (biomechanically optimal) gait pattern. By reducing the height of COM and step length, persons with PD can alter the length of pendulum (lower extremity in gait), resulting in change of its resonant frequency. Given that the resonant frequency is a primary determinant of walking speed (Holt et al., 1991), gait efficiency in persons with PD could be either maintained or improved through these biomechanical (symptomatic) modifications.

In short, a parkinsonian gait pattern on an absolute scale is energetically inefficient, when compared against the biomechanics of age-matched normal gait. But given the motor control constraints, parkinsonian gait is an energy efficient strategy that would likely lead to lower energy expenditure, if compared to age-matched controls forced to walk with a reduced step length and gait speed. The ability of a neuromotor system that has been affected by PD to make active adaptations to modulate gait patterns and accommodate unwanted variability arising from the passive gait dynamics is diminished. Slowing the gait pattern in conjunction by altering the mechanical resonance of the body would at the minimum prevent a net change in energy efficiency if it does not reduce metabolic cost.

### Tremor and rigidity

Perhaps the least intuitive, but potentially cost-saving compensatory adaptation is tremor. On the surface, one would immediately posit that tremor would require increased muscle activity, and consequently, a higher level of energy expenditure. Some would argue that tremor appears to be almost paradoxical to the hypokinetic/bradykinetic features of PD, as it is an increase in movement amplitude. Intriguingly, the severity of tremor is not necessarily related to disease progression, unlike rigidity and akinesia (Deuschl et al., 2000). Certainly, the pathophysiology of PD tremor is suggested to be different from that of the other parkinsonian features (Zetusky et al., 1985; Fishman, 2008; Zaidel et al., 2009; Mure et al., 2011). Further, parkinsonian tremor appears to be associated with abnormal neuronal synchronization in the cortico-baso-thalamic circuitry (Deuschl et al., 2000). Therefore, thalamus and subthalamic nucleus are considered as the primary targets for deep brain stimulation to suppress PD tremor. However, the direct causal relationship between parkinsonian tremor and the aforementioned central oscillators remains an enigma (Lemstra et al., 1999).

However, we believe that tremor can also be explained as a long-term compensatory behavioral adaptation to energy imbalance. Tremor is defined as hyperkinetic, involuntary and rhythmic oscillations of body segments (Deuschl et al., 2001), and it is one of the cardinal, as well as classical, motor features in PD (Zetusky and Jankovic, 1985; Zetusky et al., 1985; Farlow et al., 1993). Tremor, however, is not simply a feature of PD, but is present at rest and during postural control of the limbs in all individuals, although, most often normal tremor is generally invisible to the naked eye (Elble and Koller, 1990). In healthy individuals, the dynamics of resting tremor represent a combination of a variety of different intentional, neuromuscular, and mechanical sources, often ranging from 7 to 13 Hz (Allum et al., 1978). Parkinsonian tremor can be described as a resting tremor with a frequency typically occurring between 4–7 Hz, although tremor at higher frequencies has also been reported, especially in the early stages of the disease (Koller and Huber, 1989; Paulson and Stern, 1997; Deuschl et al., 2000). The most important factors that distinguish between parkinsonian tremor and normal resting tremor are as follows: (1) high frequency oscillations are absent in PD tremor (Vaillancourt and Newell, 2000); and (2) the power spectral density of PD tremor is narrow and concentrated around the 4–7 Hz frequency range, while power is more evenly distributed across the frequency range in normal resting tremor. By removing high frequency components of healthy tremor, there are two sources of energy cost savings that can be associated with PD. First, neuronal firing would be reduced, lowering the metabolic cost within brain structures associated with neural sources of tremor. Second, recruitment of faster and larger motor units would be reduced or possibly even eliminated. This would lead to a reduction in energy demands from fast-twitch fibers in the musculature.

The second energetic cost savings of PD tremor lies in its restriction to the mechanical resonance of the hand and arm. It is important to reiterate the energetic benefits of shifting tremor toward mechanical resonance. Movements performed at mechanical resonance exploit the inherent elastic properties of the muscle and limb dynamics to increase the “bounce for the ounce,” insofar that the need for active muscle contractions to perform the movement is reduced (Kugler et al., 1980; Kugler and Turvey, 1987; Holt et al., 1991). The ability to move at determined resonance movement frequencies (even when interacting with the environment) develops at a very early age (~8 months old), suggesting that this is an inherent component of human motor control (Goldfield et al., 1993). Moreover, even normal physiological tremor has been argued to be driven almost exclusively by mechanical factors, and a mathematical model demonstrates that physiological tremor can be generated in the absence of neurological input (Lakie et al., 2012). In addition, movements performed at resonance are also more stable and repeatable (Goodman et al., 2000). This line of evidence would indicate that parkinsonian tremor further reduces cost by exploiting the spring-like properties of the musculature to minimize the need for active contraction. Effectively, PD tremor conserves the kinetic energy by “utilizing the natural springiness” of the muscles to reduce the need for active muscle contractions to maintain posture.

One also has to keep in mind that at least some components of the tremor pattern are driven by oscillations in the CNS. If the frequency range over which the PD brain is able to transmit is limited by metabolic deficits, a possible reason why the high frequency components of healthy resting tremor is absent in PD. It is also possible that DBS therapy is particularly beneficial for the treatment of tremor, by externally reintroducing high frequency neural oscillations, minus the internal energy costs. Unfortunately, it cannot resolve problems at lower frequencies, a possible explanation as to why slower behaviors, such as gait are generally not affected positively by DBS therapy (St George et al., 2010). This would suggest that the lifting of the metabolically defined constraint on maximal firing frequency in the PD brain is required in order for all PD motor symptoms to be ameliorated.

Rigidity is another PD feature that, on the surface, does not appear to be an energy cost-saving compensatory adaptation. Rigidity is characterized as an increased resistance during a passive stretch of a muscle (Delwaide, 2001). Seemingly, rigidity appears to be an energy-wasting phenomenon since it results from uncontrolled and sustained muscle co-contraction, continuously consuming energy where muscles spend more time being “on” than “off.”

Also at the neuronal level, Takakusaki (2013) documented that increased GABAergic signal from the output channel of the basal ganglia, which is the feature of PD, could result in excessive inhibition of the brainstem muscle-tone inhibitory system, leading to muscle tone rigidity. There is little doubt that in a normally functioning neural system, excessive synchrony is clearly detrimental and wastes energy as it prevents the system from transitioning smoothly from postures to movements (Hutchison et al., 2004; Walters and Bergstrom, 2009; Avila et al., 2010), effectively “trapping” the system in place. Indeed, a healthy nervous system is able to move between the extremes of coordinated firing patterns, and high levels of synchrony are unnecessary and in fact counterproductive. Yet, in the case of PD, we are faced with neural and metabolic systems that are now inflexible (i.e., restricted to a limited number of coordinated neural firing states and metabolic energy sources). Thus, when one considers the narrow functional range with PD (i.e., reduced capacity for noise reduction and maximal firing rate), a statistical advantage of remaining in the middle becomes more economical as large-scale transitions in coordinated firing, noise levels, or firing rates are needed. Simply, because the system can no longer achieve extreme states, regressing toward the media allows the system to achieve a state of “compromise” at the neuro-metabolic level, albeit from a motor function perspective, it leads to impairments.

**Figure 3:**
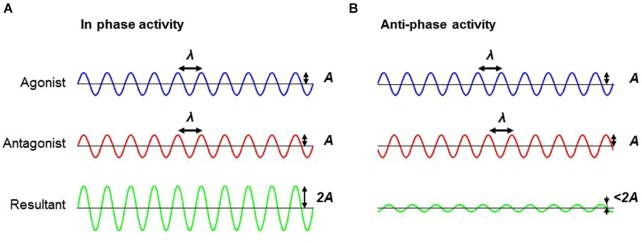
**Schematic illustrating the difference between in-phase and anti-phase firing pattern. (A)** Firing the musculature (e.g., agonist and antagonist muscles) in an in-phase pattern can facilitate an additive effect, which doubles the force amplitude with a single signal in comparison to **(B)** anti-phase firing patterns. Note the difference in the resultant amplitude between in-phase and anti-phase activation. *Abbreviations*: A, amplitude; λ, wave length.

As a result, when one considers the problem more deeply from the standpoint of muscle activation dynamics, a vastly different picture emerges. Much like holding a still posture, allowing smooth and passive motion of a limb requires two major components: (1) out-of-phase firing off the agonist and antagonist musculature, preventing co-contraction; and (2) high frequency firing patterns to prevent jerking of the limb. Rigidity is energy efficient in such a way that it reduces the firing frequency of the motor units. Another important component of rigidity is the in-phase co-contraction of agonist and antagonist muscles. Anti-phase activation demands that muscle force be signaled and generated by each muscle independently. By firing the musculature in a synchronous, in-phase pattern, energy cost savings can be realized as a single signal can now be sent to both muscles and both signals now have a facilitative effect, essentially, doubling the force output with a single signal. Indeed, rigidity is by no means functional, but is a clear reflection of the inflexibility in the PD motor system, where behaviors are limited to a narrow functional range within a framework of energy cost savings (Figure 3).

### Summary

Unfortunately, saving energy during brain activation and motor function comes at the cost of flexibility. This form of inflexibility in PD is particularly evident in a reduced ability to transition from posture to movement (and vice versa), resulting in difficulties in movement initiation and termination. What makes movement initiation difficult is the fact that voluntary movements are initiated in coincidence with the direction of the tremor (Cohen and Rosenbaum, 2007). An analogy for such a process is that movement initiation is akin to jumping onto a swinging pendulum. Normal resting tremor, due its high frequency oscillations, changes directions at a very fast rate. In addition, the lower amplitude of normal tremor reduces the need for large levels of muscle activation to overcome tremor to begin a goal-directed action. Parkinsonian tremor on the other hand, is a relatively slow and high amplitude oscillation. First, the neuromotor system has a longer waiting period in order to “catch the next wave” so that the movement can be initiated in conjunction with the direction of the tremor. The slowness of PD tremor would result in directional changes occurring with a far lower frequency. In addition, because PD tremor exploits the spring-like nature of the musculature, persons with PD have to generate a larger muscle force to overcome the “bouncing” generated by the tremor in order to start movement.

In summary, in persons with PD, it can be speculated that, to minimize energy cost, active control (e.g., lateral stabilization during gait, smooth motions of a limb, and suppression of tremor) appears to be compromised and diminished since it requires energy, and passive control (e.g., movements on the plane of progression during gait) is more inflexible since altering an automated behavior also requires energy. Hence, although mechanisms of PD tremor and parkinsonian gait do not seem to be identical (since tremor is hyperkinetic and gait is predominantly hypokinetic feature), both of them could possibly emerge from a single origin: metabolic dysfunction or energy imbalance. This speculation could be extended to difficulty in gait initiation (GI) and termination (GT) in persons with PD since both GI and GT require active control to initiate or terminate forward propulsion. Metabolic cost is required to voluntarily transition from one passively-stable condition to another. Taken together, all of these previous findings could indicate that persons with PD lack behavioral flexibility which should be necessary to adapt to environmental changes (e.g., a sudden perturbation) and/or task demands (i.e., GI and GT) since energy is required for these adaptations. It can also be postulated that this inflexibility emerged as a compensatory action for persons with PD since self-optimization of biological systems and age-related adjustment are insufficient to off-set the energy imbalance in PD.

## Hypotheses and future directions

While we outlined a new perspective on metabolic dysfunction and parkinsonian motor features in this theory-and-hypothesis paper, there are still a number of unanswered questions that are critical to understand the underlying mechanisms of features of PD and the relationship to their neural metabolic deficits. It is a particularly important avenue to explore to develop an empirical, comprehensive, and effective therapeutic strategy for this patient population.

To date, while the effectiveness of currently-available treatments, such as levodopa and DBS, is clinically accepted, and thus widely applied to a number of patients throughout decades (Deep Brain Stimulation for Parkinson’s Disease Study Group, 2001), their mechanism of their action, particularly DBS, does not appear to be fully empirically supported (Montgomery and Gale, 2008). Furthermore, while a large number of clinical studies consistently report DBS to be effective in alleviating bradykinesia, tremor, and rigidity (Deep Brain Stimulation for Parkinson’s Disease Study Group, 2001; Rodriguez-Oroz et al., 2005), it does not appear to improve postural instability and gait disturbance, and in some cases DBS worsens these symptoms over the long term (Follett et al., 2010; St George et al., 2010). The inconsistent effects of DBS in cardinal parkinsonian motor features indicate that there is more to PD than the rate model explains. These results suggest that despite the “added” DBS frequency to the underlying neural activity cannot reverse what the metabolic constraints have placed on the CNS, most likely in the realm of noise reduction (a process that requires metabolic energy). Failure to provide an effective treatment to globally improve parkinsonian motor symptoms is, at least partially, due to lack of understanding of the underlying mechanism causing PD and limitation of the current accepted model of PD (i.e., the rate model).

Here, we develop three hypotheses to test our new theoretical framework: (1) neural metabolic deficits precede the increase rate of neurodegeneration and onset of behavioral symptoms in PD; (2) motor behavior of persons with PD are more sensitive to changes in metabolic/bioenergetic state, where persons with PD will optimize their motor behavior to minimize energy; and (3) improvement of metabolic function could lead to better motor performance in persons with PD.

### Hypothesis 1—neural metabolic deficits precede the increased rate of neurodegeneration and onset of behavioral symptoms in PD

One of the major gaps in literature in PD is that the primary cause of this disorder remains unknown. Throughout this paper, we discussed neural metabolic deficits in PD, as potential key factors in its development and behavioral symptoms by way of minimizing energy for survival. Development of PD is usually a long-term process where somehow, the natural loss of DA neurons with age is accelerated, a deviation from the normal trajectory of human aging (Collier et al., 2011). Previous studies have already shown that the onset of PD precedes the motor manifestations; the pre-symptomatic phase of PD last approximately 20 years or even more (Savica et al., 2010). In fact, PD is usually diagnosed when a certain symptoms become apparent. This lag between the time when diagnosed and the actual disease onset can be also observed in a neurophysiological system: approximately a half of nigrostriatal DA neurons are lost by symptom onset (Fearnley and Lees, 1991). The fact that gradual, yet continuous, processes are central to the development of PD symptoms appears to support our hypothesis that neural metabolic deficits might well precede the increased rate of neurodegeneration and onset of behavioral symptoms in PD. To test this hypothesis, an important next step would be a prospective longitudinal (cohort) study in genetic models of PD, such as parkin knock-out mice, to determine whether metabolic dysfunction (diminished metabolic flexibility) is central to the neurodegeneration in PD. Parkin is an E3 ubiquitin-protein ligase (Shimura et al., 2000), which plays a pivotal role in mitochondrial function in such a way that it is selectively recruited to promote the autophagy of impaired mitochondria (Narendra et al., 2008). As a result, a mutation in Parkin results in increased mitochondrial aggregation and reduced autophagy of damaged or non-functional mitochondria. Taken together, loss of parkin function could eventually lead to loss of mitochondrial integrity/function in DA neurons (Palacino et al., 2004; Casarejos et al., 2006; Yu et al., 2011). Hence, parkin knock-out mice are adequate to test this hypothesis since a recent study suggest knocking out parkin in mice at adult age causes neurodegeneration in the SNc (Shin et al., 2011), while knock-out animal models of other genes, such as PINK1 (phosphatase and tensin homolog-PTEN-induced novel kinase (1), α-synuclein, LRRK2, and DJ-1 do not demonstrate any nigrostriatal degeneration (Moore and Dawson, 2008; Blesa et al., 2012).

**Figure 4:**
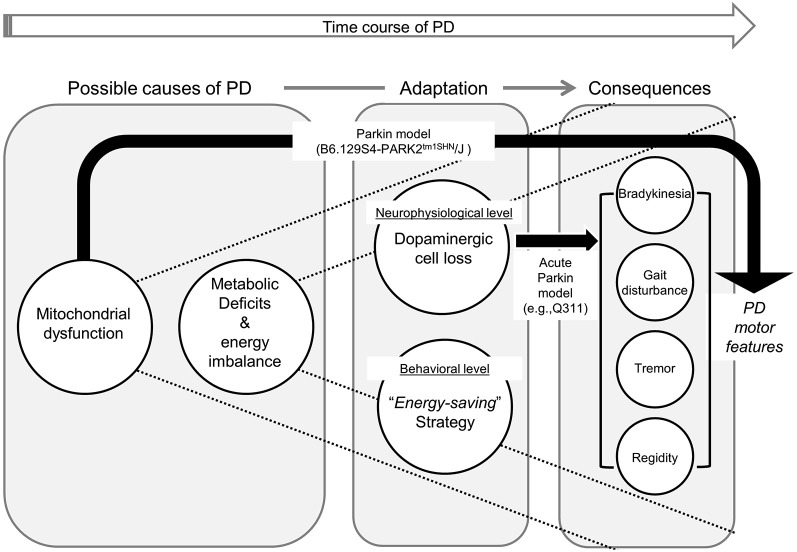
**Framework illustrating the causal relationship between neural metabolic deficits, various levels of adaptations, and consequential parkinsonian motor features**. Due to metabolic dysfunction, persons with PD continuously require neurophysiological (i.e., selective dopaminergic cell loss) and behavioral (i.e., implementation of “energy-saving” strategy) adaptations to compensate for their energy imbalance. In our proposed framework, parkinsonian features manifest as a series of long-term adaptive consequences. Black arrows illustrate how alternation in each animal PD model results in Parkinsonian motor symptoms. It is important to note that currently-available animal models cannot fully replicate PD in humans (e.g., mitochondrial dysfunction in B6.129S4-PARK2*^tm1SHN^*/J, and dopaminergic cell loss in Q411).

It is important to note, however, that not all parkin models result in DA neuron loss in SNc. While some parkin models (e.g., Q411) lead to dopamine neuron loss and behavioral changes (abnormal locomotor activation, hypoactivity, and impaired balance Lu et al., 2009), the other model (e.g., B6.129S4-PARK2*^tm1SHN^*/J) results in mitochondrial dysfunction and behavioral impairments in the absence of nigral degeneration (Goldberg et al., 2003). This conflicting result in animal model studies of PD arise from an important limitation: the progressive nature of the disorder. Without this key feature in all PD models, we cannot perfectly replicate the disease in human conditions and understand metabolic deficits, DA neuron loss, and motor symptoms are interconnected and which ones precede others in actual humans. In short, none of the existing animal models of experimental PD completely mimics the etiology, progression, and pathology of human PD (Martinez and Greenamyre, 2012). As mentioned earlier, Sotnikova et al. (2005) were able to reverse parkinsonian motor features resulting from acute dopamine depletion (e.g., akinesia, rigidity, tremor). The acute lesion or dopamine deficiency is not progressive, and in fact, many animal models, including primates, can recover from unilateral MPTP lesions (Boulet et al., 2008; Meredith and Rademacher, 2011). Moreover, PD manifests many features at various levels (cellular, structural, and functional), the aforementioned “acute” models can only partially replicate the disease (See Figure 4). Collectively, these empirical findings would suggest that the loss of DA neurons in striatum and the SNc, specifically, is not the “trigger point” of the disorder that ignites the cascade of symptoms and progressive neurodegeneration.

Comparison of the metabolic functions of both of these models should provide a clear means of testing Hypothesis 1. First, both models should demonstrate a degree of impaired metabolic function. Given that predominant use of carbohydrate (i.e., higher respiratory quotient) in aging appears to result in longevity (Rizzo et al., 2005), we should expect to see lower RQ values in both of the aforementioned parkin mouse models in comparison to age-matched controls, a process that precedes the onset of symptoms and neuron loss. This can be achieved by testing the mice in a metabolic cage. Second, a further metabolic challenge to the B6.129S4-PARK2*^tm1SHN^*/J should lead to the loss of DA neurons. Third, the loss of DA neurons in the Q411 mice should be accelerated by a further metabolic challenge.

The strongest empirical outcomes would arise from experiments where the symptoms of PD can be induced in normal control mice through manipulations of metabolic function over a prolonged period of time *without* the need for lesions to the SNc. For example, Bassil et al. (2014) have proposed that GLP-1 (glucagon-like peptide-1) is deficient in PD and other neurodegenerative disorders, while IGF-1 (insulin-like growth factor-1) levels are excessive. A good test of Hypothesis 1 would be an experiment where IGF-1 levels are increased and GLP-1 actions are blocked at different points in the animal lifespan. If the hypothesis is correct, these animals will exhibit PD symptoms later in life.

In Sum, if this hypothesis holds true, our theoretical framework would suggest investigating underlying mechanisms causing the metabolic dysfunction and energy imbalance is vital to further understand the primary cause of PD, rather than subsequent DA cell loss. Ultimately, this approach could help developing a new diagnostic tool for: (A) early detection of PD; and/or (B) better PD risk-factor determination.

### Hypothesis 2—motor behavior of persons with PD are more sensitive to changes in metabolic/bioenergetic state

Knowing that PD motor symptoms are adaptations to underlying metabolic dysfunction, then there is the possibility that the motor symptoms can be exacerbated by changes in metabolic state and that inability to implement proper behavioral adjustments corresponding to these changes could result in aggravated fatigability. In fact, fatigue is one of the major and most common non-motor problems in persons with PD, approximately 44% of PD patients reported subjective fatigue (Karlsen et al., 1999). In addition, individuals with PD are also more easily fatigued during motor tasks (i.e., decay of maximum force production relative to individuals’ peak force throughout muscle activation) than healthy counterparts during sustained maximum isometric forearm flexion task (Ziv et al., 1998). These findings demonstrate the potential link between metabolic state and motor function. Building on the aforementioned studies, further evidence of the link between metabolism and motor function in PD could be improved by additional physiological measurements. For example, blood lactate assays could be obtained, or measures of oxygen consumption and respiratory quotient could be introduced. These physiological measurements would yield further insight into the metabolic state of the individual during the fatiguing task, that could be in turn be correlated with motor patterns.

Other tests that provide perturbations to metabolic state could also be employed. As another example, the test of metabolic flexibility, i.e., a hyper-insulinemic clamp could be used to alter RQ in human subjects. Subjects could then undergo motor testing, e.g., gait, tremor, etc. One would thus predict that the hypo- and hyper-kinetic features of PD will be affected by the change in metabolic state, but not in healthy controls. There are other potential manipulations to metabolic state through dietary factors and nutritional supplements.

### Hypothesis 3—improvement of metabolic function could lead to better motor performance in persons with PD

If the aforementioned two hypotheses hold true, we need to consider changing the course of designing effective therapeutic options for this population: from finding optimal symptom-specific interventions (e.g., walking faster, regaining balance and reducing tremor amplitude) to new approaches focusing on metabolic/bioenergetic restoration and improving metabolic flexibility. These approaches are based on cumulative evidence from both animal and postmortem human brain tissue, implicating mitochondrial dysfunction in PD (Beal, 2005; Lin and Beal, 2006; Chaturvedi and Beal, 2008, 2013). Moreover, Morais et al. (2014) recently investigated the link between energy deficits and PD using mice and fruitflies with a defective PINK1 gene and demonstrated that the defect resulted in a specific loss of phosphorylation in Complex I, which is associated with decreased energy production. Intriguingly, however, they also showed that Complex I deficits and energy production could be rescued in cells derived from patients with PINK1 mutations after restoring correct phosphorylation of Complex I, and that parkinsonian symptoms consequently decreased or disappeared. Effectively, these aforementioned studies raise the possibility that rescuing a genetic component of mitochondrial dysfunction and/or restoring energy production can play a crucial role in improving symptoms in this population. Indeed, mitochondria targeted therapeutic interventions has recently gained attention and research evaluating its effect has begun in neurodegenerative disorders, such as PD and Huntington’s disease (Chaturvedi and Beal, 2013).

One mitochondrial bioenergetics agent studied in previous literature of PD is creatine, which is involved in energy supply to the muscle and nerve cells. Briefly, creatine (or phosphocreatine (PCr) can serve as a temporary ATP source via a reversible reaction where PCr transfers its phosphate to ADP to form ATP (Andres et al., 2008). Given that a previous study showed that creatine exerted a neuroprotective effect on DA neurons of the SN and depletion of dopamine level in a MPTP induced mouse model (Matthews et al., 1999), it can be speculated that creatine supplementation can improve impaired metabolic function in persons with PD, resulting in protecting DA neurons requiring high energy for survival (as previously mentioned). Furthermore, a growing body of literature shows promising benefits of creatine in improving various parkinsonian symptoms, such as muscle endurance (Hass et al., 2007), progression of overall UPDRS score (Ninds Net-Pd Investigators, 2006), and individuals’ mood (Bender et al., 2006).

In addition, PD has been shown to result in increased IGF-1 levels, indicative of insulin resistance (see Bassil et al. (2014) for a review). Data from a small cross-sectional sample found elevated IGF-1 levels to be correlated with PD symptom severity as measured through Hoehn-Yahr (Numao et al., 2014). Hence, another alternative therapy would be the use of medications that improve insulin sensitivity, such as metformin, which has now been shown to have a neuroprotective effect on the MPTP mouse model of PD (Patil et al., 2014). IGF-1 levels can be lowered and insulin sensitivity increased through treatment with the growth hormone receptor antagonist, pegvisomant (see van der Lely and Kopchick (2006) for a review). Further research in this area is needed, in conjunction with measures of nigrostriatal dopamine neuron loss in order to test the hypotheses beyond that of motor symptoms.

## Concluding remarks

Unfortunately, there is currently no cure for PD; thus currently-available treatment options only focus on alleviating and delaying motor symptoms associated with PD. Although much progress has been made to develop and evaluate various treatment options for this population, including but not limited to DA medications, deep brain stimulation, and physical activity-based interventions (resistance training, treadmill training, Tai Chi, etc.), the optimal intervention for this pathological population has to be yet explored. Instead, there is even contradictory evidence in a recent mice model study suggesting that exercise does not slow down progression of symptoms, but even accelerate their progression or onset of disease in neurological disorders. Specifically, mice with HD in pre-symptomatic stage completing voluntary wheel had an earlier onset of HD symptoms and reduced striatal volume and motor impairments when compared to non-runners (Potter et al., 2010). They claimed that exercise could be detrimental to their vulnerable nervous system; thus providing exercise regimens to patients with neurodegenerative disease should be done carefully.

Taken together, instead of conventional exercise modalities with little or no benefits, or even adverse effect to symptoms, another potential approach to improve parkinsonian symptoms could be alleviating mitochondrial dysfunction and associated bioenergetics deficits. Because of this decisive involvement of mitochondrial dysfunction and its associate bioenergetic defects, enhancing normal mitochondrial function and/or improve energy imbalance may be beneficial to improve debilitating symptoms in PD. Effectively, evidence to date ranging from molecular to behavioral studies lead us to propose the third hypothesis that improvement of metabolic function/flexibility could lead to better motor performance in PD. To test this hypothesis, it is imperative to include measurements/calculations of their metabolic function and/or energy expenditure and examine the relationship between the improvement in metabolic function and the one in motor behavior when evaluating any therapeutic interventions.

We have presented a new theoretical framework that argues that underlying neural metabolic deficits are central to the development of PD motor symptoms. This framework in conjunction with the testable hypotheses proposed in this paper will provide a novel viewpoint that can elucidate the connections between metabolic, neural and motor function in PD. Ultimately, we hope this theory-and-hypotheses paper will lay the foundation for future clinical and therapeutic research to design a precise diagnosis and effective therapeutic interventions on the basis of the corrective knowledge obtained from interdisciplinary field of sciences, including molecular biology to behavioral sciences.

## Conflict of interest statement

The authors declare that the research was conducted in the absence of any commercial or financial relationships that could be construed as a potential conflict of interest.
